# Multi-step atomic mechanism of platinum nanocrystals nucleation and growth revealed by in-situ liquid cell STEM

**DOI:** 10.1038/s41598-021-03455-w

**Published:** 2021-12-14

**Authors:** Walid Dachraoui, Trond R. Henninen, Debora Keller, Rolf Erni

**Affiliations:** Electron Microscopy Center, Empa––Swiss Federal Laboratories for Materials Science and Technology, Überlandstrasse 129, 8600 Dübendorf, Switzerland

**Keywords:** Chemistry, Materials science, Nanoscience and technology

## Abstract

The understanding of crystal growth mechanisms has broadened substantially. One significant advancement is based in the conception that the interaction between particles plays an important role in the growth of nanomaterials. This is in contrast to the classical model, which neglects this process. Direct imaging of such processes at atomic-level in liquid-phase is essential for establishing new theoretical models that encompass the full complexity of realistic scenarios and eventually allow for tailoring nanoparticle growth. Here, we investigate at atomic-scale the exact growth mechanisms of platinum nanocrystals from single atom to final crystals by in-situ liquid phase scanning transmission electron microscopy. We show that, after nucleation, the nanocrystals grow via two main stages: atomic attachment in the first stage, where the particles initially grow by attachment of the atoms until depletion of the surrounding zone. Thereafter, follows the second stage of growth, which is based on particle attachment by different atomic pathways to finally form mature nanoparticles. The atomic mechanisms underlying these growth pathways are distinctly different and have different driving forces and kinetics as evidenced by our experimental observations.

## Introduction

Understanding crystal growth mechanisms and related atomic structure evolution plays a fundamental role in controlling the size, shape and crystallinity of nanocrystals. This knowledge is essential for tailoring their properties and optimizing their performance as well as for engineering new nanomaterials for different applications^1–3^. Theories on growth mechanisms suggest two categories: classical mechanisms where the growth by monomer attachment is considered as the main mechanism for nanoparticle growth, and non-classical mechanisms related to particle-mediated growth and particle assembly^4–12^. Classical Ostwald ripening (OR) is often used to explain and describe the crystal growth, involving the growth of larger particles at the expense of smaller ones, according to the Gibbs–Thomson relation^13–15^. Different from OR and monomer attachment, particle growth by oriented attachment (OA) refers to another mechanism in which nearby particles with pre-aligned crystallographic orientations coalesce to form larger single particles^16–18^. To clarify the classical and non-classical growth mechanisms, several studies have been realized to image crystal growth at the nanometer and/or atomic scale by in-situ liquid-phase and cryo transmission electron microscopy (TEM)^19–25^. One particular study conducted by Zheng et al. aimed at observing the growth trajectories of single colloidal platinum nanocrystals and showed that the Pt nanocrystals can grow either by monomer attachment from solution or by coalescence^22^. Real-time observation of CaCO_3_ nucleation has been reported by Yuk et al., showing that multiple pathways occur simultaneously^26^. Zheng et al. conducted an in-situ TEM study to image in real-time the growth of Pt_3_Fe nanorods in solution from nanoparticle building blocks via attachment, followed by straightening, orientation adjustment and shape correction, to eventually yield single-crystal nanorods^24^. All these experimental observations clearly show that realistic nucleation and particle growth reactions are more complex than what classical theory predicts.

Yet, despite all these valuable studies, the exact atomic mechanisms of growth starting from the very early stage (nucleation) remains unclear. For instance, it is unclear how and when growth via particle attachment takes place and when instead particle growth via monomer attachment occurs. How is the particle growth initiated at all? Does the nucleation process proceed by nanoparticles attachment, analogous to conventional growth by monomer attachment at the atomic-level?

From an experimental point of view, observations at atomic resolution are highly challenging as these early events of particle growth occur rapidly. Recent advances in high-speed detectors and data generation and processing have greatly improved the attainable temporal resolution in electron microscopy. Moreover, recent developments in transmission electron microscopy and microfabrication provide opportunities for imaging particles in a liquid environment^27–41^. However, silicon nitride-based liquid cells limit the attainable spatial resolution due to the thickness of the silicon nitride membranes (around 50 nm) added to the thickness of encapsulated liquid phase ranging from 50 nm to 5 µm. Alternatively, non-commercial graphene liquid cells (GLCs) provide the opportunity to observe nanoparticles at the true atomic-level. Graphene sheets are composed of a few graphitic monolayers with an overall thickness below 1 nm, allowing for imaging liquid samples and particles therein at Ångström level. The impermeability and the mechanical flexibility of graphene enable the entrapment of liquid nanoreactors without risk of leakage to the surrounding vacuum environment of the electron microscope. Moreover, the chemical neutrality and electrical conductivity of graphene enable the study of chemical reactions inside GLCs while minimizing unwanted irradiation effects^42,43^.

In this work, we demonstrate that using GLCs combined with aberration-corrected scanning TEM (STEM) with a fast acquisition strategy substantially improves the spatial and temporal resolutions for reactions in liquid phase and allows us to study the exact growth mechanisms of Pt nanoparticles at the atomic-level from metastable states to final crystals. Without loss of temporal and spatial resolution, we unravel the complete chronology of Pt NPs formation in aqueous solution to unify the non-classical nucleation theory and the classical nucleation theory in order to explain the nucleation-and-growth process of NPs in conditions similar to those in lab-synthesis conditions. Most importantly, our ability to follow atom-by-atom the multi-step mechanism of growth allowed us to shed light on the atomic mechanism of the intermediate steps such as the amorphous-to-crystalline transition and the defect elimination in the second stage of growth. We found that the critical size of amorphous clusters that are able to overcome the energy barrier to transform to the crystalline phase is around 1 nm. In comparison to the previous studies of growth mechanisms using in situ TEM, our study focuses at atomic scale on the intermediate steps in both first and second stage of the multi-step growth mechanisms. Interestingly we found that the transition from amorphous to crystalline phase is not a sudden transformation, however this transformation itself is a continuous improvement of crystallinity catalyzed by atomic attachment and exchange. In the second stage where the nano-classical theory dominates the growth, we found in most cases that the attachment of particles is followed by a defect elimination processes giving rise to a well crystalline final nanocrystal.

## Results

We use 5 mM aqueous solution of Na_2_PtCl_4_·2H_2_O to study nucleation and growth of Pt nanoparticles. The radiolysis of water by the high-energy electron beam results in the creation of well-established primary products: hydrated electrons (e_h_^−^), hydrogen radicals (H^·^), hydroxyl radicals (HO^·^), hydrogen peroxide (H_2_O_2_), hydronium ions (H_3_O^+^), hydroperoxyl radicals (HO_2_^·^), and hydrogen (H_2_). Strong reducing agents, such as e_h_^−^ and H^·^, react with the complex ions of the Pt precursor at diffusion controlled rates. Uniformly distributed within the irradiated area, these agents reduce the Pt ions to zerovalent metal atoms or atomic clusters^44^. The reduction of the encapsulated Pt precursor by the electron beam thus triggers nucleation and growth of Pt nanoparticles. One should keep in consideration that electron-beam induced heating could change the local temperature, which has an effect on the radiochemistry inside the aqueous solution in the liquid cell^45–47^. However, the temperature increase inside the GLC remains unknown and cannot be measured directly in our case. Since the aqueous solvent remains liquid and does not form gaseous pockets, we must conclude that the temperature increase in the liquid pocket is moderate and that it is the radiochemistry of the electron beam that governs the observed reaction. Moreover, the graphene windows and the small volume warrant that possible heat can be dissipated quickly.

**Figure 1:**
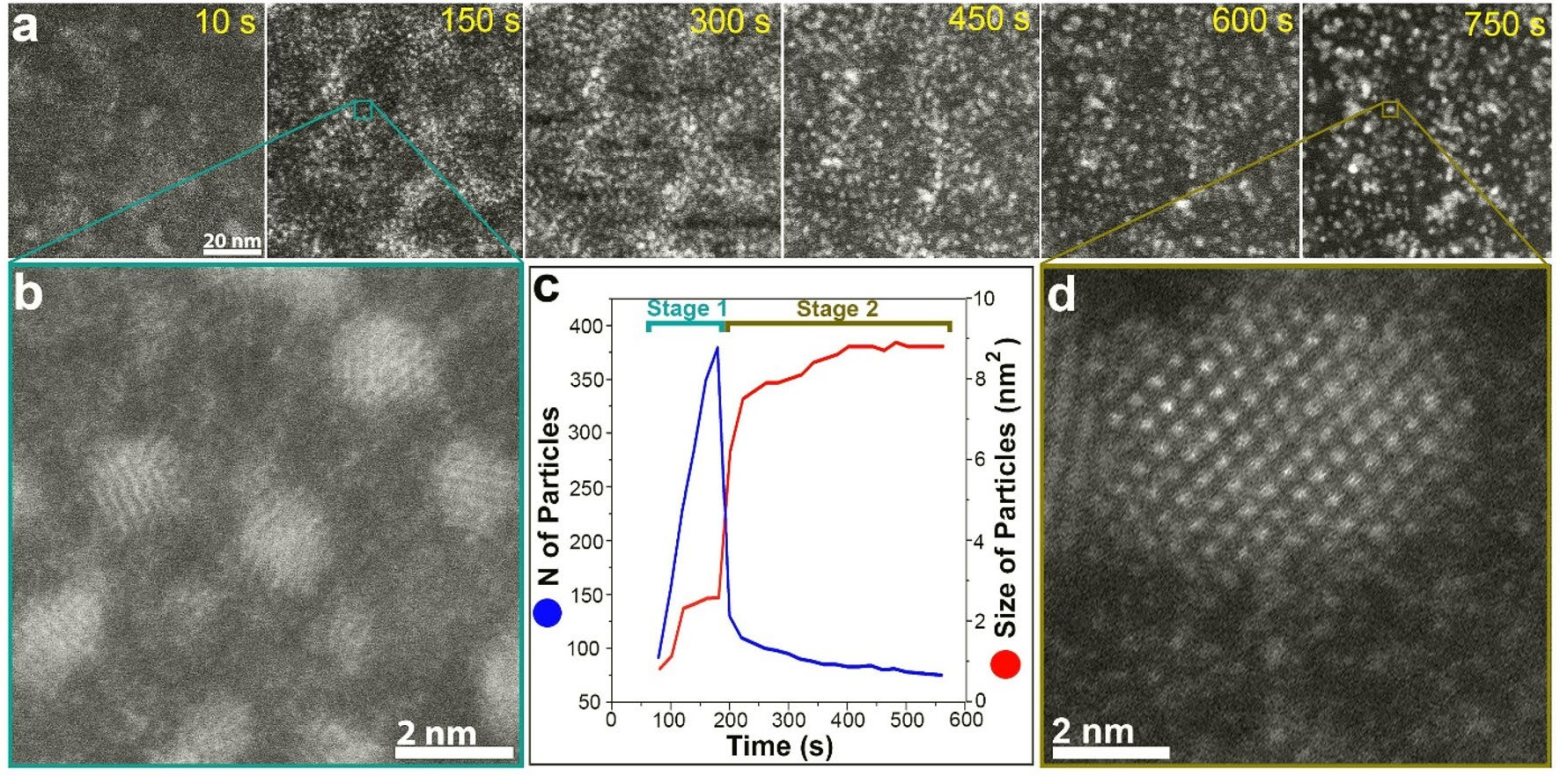
The growth of Pt nanoparticles in a GLC during exposure to the electron beam. (**a**) Sequential ADF-STEM images showing the real-time growth of Pt nanoparticles via two stages. (**b**) High-resolution ADF-STEM image of nanoparticles formed during the first stage (nucleation and monomer attachment). (**c**) Average particles size evolution (red) and the number of particles (blue) as a function of time. (**d**) High-resolution ADF-STEM image of a particle formed during the second stage (nanoparticle attachment). Images are shown in false colour.

Figure 1 shows a sequence of annular dark-field (ADF) STEM images depicting the growth of Pt nanoparticles (also see Movie S1 for details). Two distinct stages can be identified. During the first growth stage, many small nanoparticles are formed when the electron beam reduces the Pt precursor. The second stage of growth is dominated by nanoparticles interacting with each other to form bigger nanoparticles with different shape and size (also see Movie S2 and Fig. S2 for details).

Our real-time observation shows clearly the existence of single particles at the very beginning with a size less than 2 nm (clearly visible in the zoom-in of the zone marked with the green square: Fig. 1b). As the growth proceeds, small aggregates become dominating. High-resolution ADF-STEM images and the analysis of their Fourier transforms (FT) show the presence of a face-centered cubic (fcc) Pt nanocrystal with a high degree of crystallinity and a lattice constant of about 0.39 nm, similar to the bulk value of Pt^48^. The size and number of nanoparticles as a function of time (red and blue successively) are represented in Fig. 1c. During the first growth stage, the number of particles gradually increases and reaches a maximum at t ~ 180 s, after which, suddenly, the number of particles drops significantly while the average particle size increases. Therefore, the growth process enters the second growth stage at about 180 s, where the sudden decrease in the number of particles and the correlated increase in particle size is mainly due to the coalescence of individual particles.

**Figure 2:**
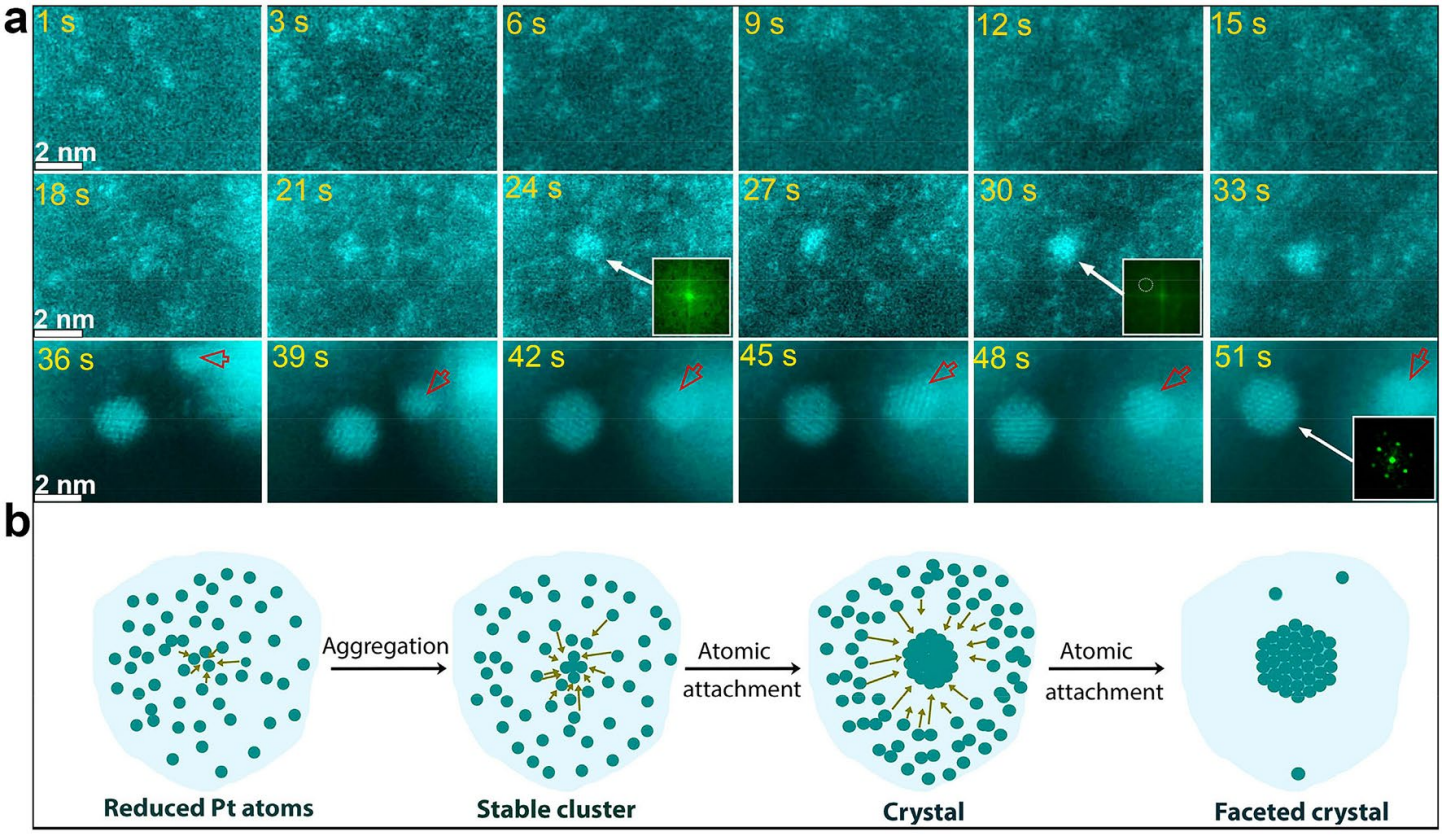
Nucleation and growth of nanoparticles via a two-step nucleation mechanism, followed by atom-by-atom attachment. (**a**) Image sequences from Movie S3 showing the nucleation and growth of Pt nanoparticle via atom-by-atom growth, where atoms migrate through the liquid to attach to an initially formed cluster. At the end of the series, a single crystal of about 2 nm diameter is formed. (**b**) Schematic illustration of the first growth stage via atom-by-atom nucleation and growth. Images are shown in false colour.

Distinguishing these two stages of particle growth, the very early stage of growth was monitored using high-resolution ADF-STEM images with a temporal resolution of 2 frames/s, made possible by the very thin liquid nanoreactors (less than 50 nm) and the fast acquisition scheme. Figure 2a shows sequential atomic-resolution snapshots of the nucleation of a selected Pt nanoparticle, using a false-colour representation to highlight the Pt-rich areas. The time *t* = 0 s in the Movie S2 and in Fig. 2a represents the starting point of imaging in that particular area, after having optimized the imaging conditions in a different area to minimize the beam effect in the area of observation. The electron dose and the imaging conditions were optimized to enable the growth of Pt nanoparticles with kinetics suitable to track the nucleation without inducing unwanted beam effects. After irradiation with the electron beam, with an electron dose rate of about 4.2 × 10^3^ electrons/Å^2^s, the Pt precursor is reduced. Here the brighter dots in the imaged area in Fig. 2a correspond to reduced Pt atoms. After 18 s, the Pt atoms start aggregating to form a stable cluster. Then, atoms from the surrounding liquid rapidly attach to the nanocluster acting as seed. The additional atoms are delivered to the seed by random collisions. During this atomic attachment, the region surrounding the seed nanocluster becomes darker, indicating that the direct environment of the growing cluster becomes depleted in Pt. This depleted region continues to expand while the nanocrystal increases in size (also see Movie S3 for details). Using the FTs of the images, we can follow the evolution of the crystallinity of the nanocrystal. The FT patterns in the insets in Fig. 2a show that the formed cluster is initially amorphous until t = 30 s, when it reaches a diameter of around 1 nm it becomes stable and a first reflection appears that indicates crystallinity (highlighted with a white circle in the FT inset, Fig. 2a) and which matches with the (200) lattice plane spacing of fcc Pt. This is consistent with the two-step nucleation mechanism, where initially formed stable or metastable precursor can proceed to crystallize into a crystalline phase by overcoming an energy barrier. The formed ordered stable species never dissociate and can crystallize by monomer addition^49^. Indeed, in our study the crystallinity of the Pt nanocluster continues improving over the rest of the growth period until a distinct single crystal is formed with Pt atoms adopting uniform inter-atomic distances. The single-crystallinity of the fcc structure is reflected in the atomic–scale image (t = 51 s) and the corresponding [111] FT pattern of cubic platinum (see Movie S3 and Fig. S3 for details). The Pt nanocrystal subsequently rotates or transforms, displaying different orientation from 37 to 51 s, then rotates or transforms again to the [111] orientation. By evaluating the intensity of spots corresponding to crystalline Pt in the FT patterns (Fig. S3), the degree of crystallinity can be estimated showing an abrupt increase of the atomic ordering in the Pt nanocrystal during heterogeneous nucleation showing that crystallization of Pt has occurred from an amorphous cluster. The first growth stage, described here by nucleation and formation of the Pt nanocrystal, follows a typical two-step nucleation pathway, where in the first step an amorphous cluster is formed from a supersaturated solution and in the second step, the cluster reorganizes into an ordered structure by overcoming the energy barrier. These two nucleation steps have been initially proposed for crystallization reactions of proteins and recent experimental and theoretical studies suggested that this mechanism may underlie most of the crystallization processes from solution^50,51^. Previous work has demonstrated that energy transferred from the electron beam can influence, melt and even vaporize the different metal nanocrystals^52^ and recently Cao et al. demonstrated that under the influence of the electron beam, amorphous Au nanocrystal had enough energy to overcome the energy barrier to crystallization^44^. Therefore, we can conclude that Pt clusters of around 1 nm had enough energy from the electron beam to overcome the energy barrier to crystallization.

During the above-described nucleation process a clearly visible migration of atoms from solution to the nanocrystal occurs, leaving behind a surrounding region of dark contrast, which is due to Pt depletion. After Pt depletion of the direct environment, the growth via atom-by-atom process ceases and the size of the nanocrystal stops increasing. Figure 2b shows a schematic illustration detailing the first growth stage of a Pt nanocrystal, via atom-by-atom attachment following a two-step nucleation pathway, starting from single reduced Pt atoms to a well-crystallized nanoparticle.

While the previous observation focussed on the nucleation and initial growth of a selected Pt nanoparticle, a second crystal formed similarly in the same observation area following the same mechanism (highlighted with red arrow in Fig. 2a). This second particle will be important to document the second stage in the growth process.

### Growth by oriented attachment (OA)

**Figure 3:**
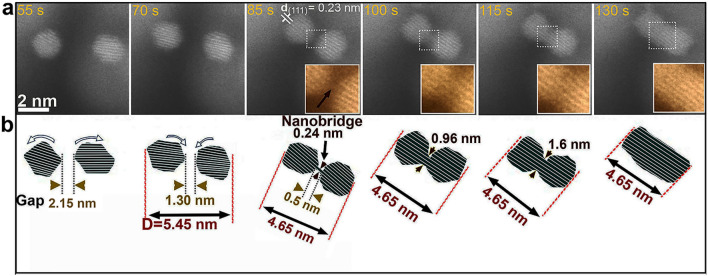
Oriented attachment via nanobridge-induced contact. (**a**) Atomic resolution ADF-STEM time-series showing the OA process of two small Pt nanocrystals, indicating the approach of nanocrystals and pre-alignment by rotation, followed by nanobridge formation (marked by the black arrow in the inset at 85 s) and subsequent fusion of the nanocrystals. (**b**) Schematic illustration of the nanobridge-induced OA process. Images in the insets are shown in false colour.

The same nanocrystals, previously described in Fig. 2, grown *vi*a atom-by-atom mechanism in the first growth stage, start, after monomer depletion of the environment, a second growth stage by attachment-based crystal growth, where they start to interact with each other to form a larger nanocrystal. The time-resolved high-resolution ADF-STEM images in Fig. 3a show that the pair of nanoparticles, formed during the first growth stage (Fig. 2a), undergoes oriented attachment in the second growth stage. Here, during the whole process the two particles are in the same focus, which allows us for considering the projected distance between the particles as the real separation distance. Initially, both nanoparticles of diameters of about 2 nm are separated by about 2.2 nm at t = 55 s. Then, both particles start to continuously move and rotate in apparently random fashion in the solution, while slowly approaching each other (see Movie S4 for details). When they get closer, to a distance of about 1.5 nm, the Pt particles start to rotate in opposite directions as is shown with blue arrows in Fig. 3b (clockwise for the left one and anti-clockwise for the right one) until their {111} facets are perfectly aligned. When the particles are separated by a gap distance of around 0.5 nm and their {111} crystal facets are perfectly aligned, OA is accomplished via formation of a nanobridge, marked by the black arrow in the inset in Fig. 3a at t = 85 s. The formation of the nanobridge is different from the jump-to-coalescence mechanism that alternatively can trigger particle coalescence (see next section). To reveal the exact atomic mechanism of the nanobridge formation process, high-resolution ADF-STEM images were acquired in liquid mode at the interface between a pair of Pt particles during the rapid bridge formation. When the particles are about 0.5 nm apart from each other, a nanobridge with clear lattice fringes of one atomic layer thickness forms across the gap (marked by a black arrow in the inset in Fig. 3a at t = 85 s) before full contact is achieved. This observation is consistent with previous work where Au atoms nucleated between merging nanocrystals and formed a thin nanobridge. Then the thickness of the nanobridge increases^53^. Our experimental observations are detailed in the schematic representation in Fig. 3b: the nanobridge marked by the red triangle starts with a width of about 0.24 nm and eventually reaches a width of 1.6 nm. Thereafter, the pair of nanocrystals completely merges to form one single crystalline nanoparticle. Therefore, this mechanism is in agreement with the mechanism suggested by Tang et al. that describes the bridge-induced contact and fusion of gold nanoparticles. However, their description of this mechanism points to a general coalescence process^54^, while in our case the bridge-induced coalescence via OA is detailed on the atomic-level.

### Growth by imperfect particle attachment (jump-to-coalescence)

**Figure 4:**
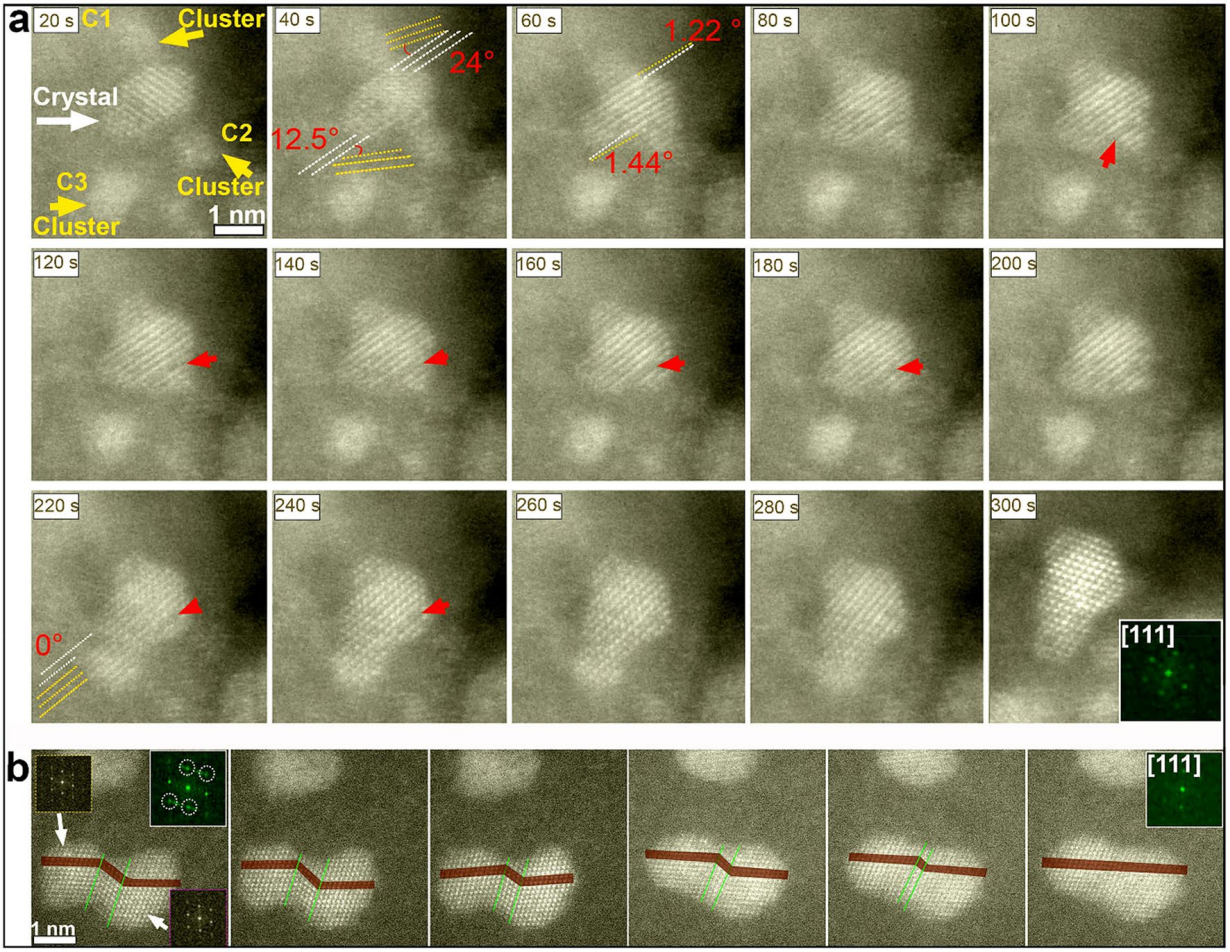
Imperfect nanocrystal attachment. (**a**) Two ultra-small nanocrystals (designated by C1 and C2) approach a larger stable crystal with an initial misalignment angle of 24°, and 12.5° respectively. Then the nanocrystals rotate to reduce their crystallographic misorientation in respect to the larger crystal. Then, still with a small misalignment angle (of 1.22° and 1.44° respectively) the nanocrystals merge with the larger crystal within an imperfect coalescence. However, such imperfect coalescences induce defects as highlighted by the red arrows. Finally, a third nanocluster C3 undergoes OA to the larger nanocrystal at the end of the observation time. (**b**) Two Pt nanocrystals involved in an imperfect attachment process inducing twin boundaries. The twin boundaries are indicated by green lines. The twins are removed in a continuous process until a monocrystal is formed (confirmed by the inset FT pattern). Images are shown in false colour.

In contrast to the OA shown above, occurring for particles with similar sizes, we also recorded another type of attachment typical for nanoparticles larger than about 1 nm merging with nanoparticles of around 1 nm and/or with smaller clusters, called imperfect particle attachment. The smaller particles attach to the stable, larger nanoparticles by a coalescence process, followed by a rearrangement process, to eventually form a larger single crystal after undergoing a defect elimination processes. Figure 4a shows real-time high-resolution ADF-STEM images documenting the coalescence of three clusters (around 1 nm) with a larger nanocrystal (more than 2 nm in size) that is stable and well-crystallized (more details in Movie S5). In this movie, the first two, clearly crystalline, small nanoparticles (designated by C1 and C2 in Fig. 4a) move closer to the bigger crystal. Once the two clusters approach the large crystal, with initial misalignment angles of 24° and 12.5° for C1 and C2 respectively, a narrow neck is formed, made of densely packed {111} planes. The misorientation angle of both particles is then gradually reduced by rotation. These imperfect crystallographic alignments give rise to defects such as dislocations, twin boundaries and stacking faults (red arrows in Fig. 4a). Figure S4 shows additional details about how the defects can be formed owing to imperfect attachment. After attachment of the smaller particles, the larger particle starts a relaxation process to remove the defective areas, until a perfect monocrystalline nanoparticle is established (Fig. 4a at t = 300 s) as confirmed by the inset FT showing consistent sharp spots. It appears that, under the present experimental conditions, structural rearrangements of the nanocrystal after imperfect attachment occurred not only via surface rearrangements but also via reorganization of the complete nanocrystal interior. Interestingly, unlike oriented attachment, Pt nanocrystals with different sizes including clusters of around 1 nm are involved in the growth by imperfect attachment.

Figure 4b shows a representative example of a nanocrystal grown via an imperfect attachment of two particles of around 2 nm in size. Two twin boundaries are clearly visible at the interface and they are also confirmed by the FT pattern in the inset, where a doubling of the reflections indicates the presence of twin boundaries (highlighted with white circles). After coalescence, the particle starts a relaxation process to remove the twinned area between the crystals via thinning of the twinned area. This process is illustrated in Fig. 4b, where the twin boundaries are outlined by the green lines (see also Movie S6 for more details). Despite the processes of defects elimination in imperfect attachment growth based mechanism.

### Growth by monomer attachment and Ostwald ripening

**Figure 5:**
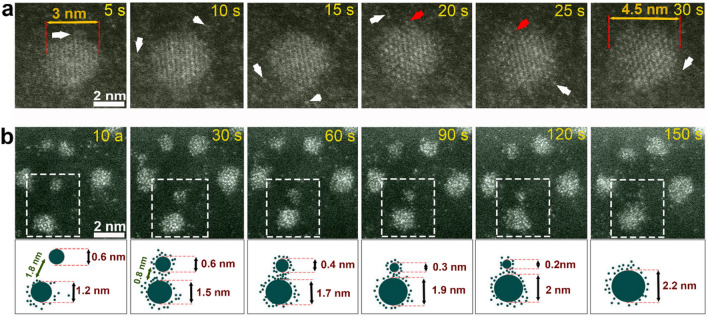
Nanocrystal growth by monomer attachment and Ostwald ripening. (**a**) ADF-STEM time-series showing a particle growing by monomer attachment, where visible, attachment of free Pt atoms (white arrows) form new atomic layers on top of the old ones (red arrows). (**b**) ADF-STEM time-series showing a particle growing by Ostwald ripening, where single atoms and clusters migrate through liquid from the smaller particle (top) to the larger particle (bottom). In the bottom, schematic illustration of the Ostwald repining process. The yellow arrows highlight the single atoms and clusters detached from the smaller particle. Images are shown in false colour.

The addition of single Pt atoms and/or small clusters also continues in the second growth stage as a growth process parallel to the coalescence processes. Figure 5a shows a sequence of high-resolution ADF-STEM images of one particle growing via monomer attachment. During the growth process, a Pt nanocrystal of around 3 nm diameter serves as seed, where the additional Pt atoms are delivered by random collisions (white arrows in Fig. 5a). Consequently, the size of Pt nanocrystal increased to reach 4.5 nm at the end (Fig. 5a). The red arrows in Fig. 5a highlight the formation of new atomic layers during the particle growth. Interestingly, many particles have been observed to dissolve back into the precursor solution (see Fig. S5 for details). This means that the Pt atoms attaching onto existing particles may stem either from the reduced precursors, from smaller particles that dissolve back into the surrounding liquid, or from particles that undergo Ostwald ripening. Indeed Ostwald ripening has frequently been observed to occur simultaneously with other growth pathways^55^. Figure 5b shows a series of ADF-STEM images of two nanoparticles, a larger and a smaller one, which are involved in an Ostwald ripening process (see Movie S7 for details). The smaller particle (top) moves inside the liquid until it gets close to the bigger particle as it is shown in the schematic in Fig. 5b, then releases single atoms (e.g. green small dots in the schematic description of Fig. 5b) which migrate through the liquid to the larger particle on the bottom which increases in size while the size of the smaller particle decreases. During the whole observation period, atoms are transferred from the small particle to the larger one until its entire mass is completely transferred to the larger one.

Compared to the previously described growth processes (i.e. coalescence based growth), which lead to significant and abrupt shape and size changes, the growth via monomer attachment and Ostwald ripening maintains nearly a constant shape with no sudden size and shape changes.

In summary, the present work focusses on unravelling the atomic mechanisms that govern nucleation and growth of Pt nanoparticles. We show real-time observations of the earliest stages of Pt nanoparticle nucleation in a supersaturated aqueous solution at atomic-level. Our data reveal that nucleation of Pt nanoparticles from the aqueous solution does not proceed via the classical pathway but follows more complex routes, where the particles nucleate via a two-step process. The first nucleation step is the formation of a cluster from reduced Pt atoms in the supersaturated solution. The second nucleation step is the reorganization of the cluster into an ordered structure by overcoming the energy barrier to crystallization, where a well-defined nanocrystal is formed via monomer attachment. Interestingly, our study documents that this two-step nucleation process is the first stage of two main growth stages, where the second growth stage is dominated by crystal attachment or coalescence mechanisms. The second stage starts once the nanoparticles’ environment is depleted of reduced Pt atoms. Different growth mechanisms were observed to occur simultaneously during this stage, such as oriented attachment, imperfect attachment, monomer attachment and Ostwald ripening. The exact atomic mechanisms of each growth process are revealed, showing that particles are involved in complex routes to form larger, well-defined nanocrystals. Oriented attachment, Ostwald ripening and monomer attachment directly give rise to well-defined single crystals. However, the imperfect attachment growth route induces defects, such as dislocations and twin boundaries, where an extra step of defect annihilation is observed before a well-defined single crystal is formed. This study shows the ability of using GLCs to continuously follow at atomic level the complete growth of NPs. Our observations allows us to show the diversity and the complexity of nanoparticle growth processes in liquid. We were able to show that at atomic level the mechanisms controlling and triggering particle growth can be uncovered atom-by-atom in lab-synthesis conditions. Our observation reveal that diverse growth pathways proceed simultaneously. This is in contrast to the study made by Cao et al. for example, where at atomic level particle growth is investigate that occurs strongly confined in single-walled carbon nanotubes^56^. Our observation at atomic level in aqueous solution not only confirm mechanisms suggested by theory and unify the classical and non-classical theories, we also identified unexpected growth phenomena and more intricate coalescence events such as the role of defects in the growth as well as their elimination processes. In comparison to our previous study in the atomic mechanisms of gold nanoparticles growth in ionic liquid^57^, this study is more close to the synthesis of materials in real lab-conditions and develops on the second stage in term of atomic mechanisms such as the difference between Ostwald ripening and oriented attachment. Similarities between the two studies of Pt and Au, however, clearly reveal a trend to generalization that particle growth processes are more complex than captured by classical theory. The continuous atom-by-atom imaging of nanomaterials growth from the very earlier stage to the final stage in aqueous solutions in addition to the ability to image the simultaneous atomic phenomena of each stage is unpreceded. Moreover, our observation reveal the exact atomic mechanisms and the driving forces of the transition from monomer attachment induced first stage of growth to nanoparticle attachment induced second stage of growth. An interesting point of our study concerns the transition from amorphous to crystalline state, which we show is not a sudden transformation but by itself a continuous improvement of crystallinity catalyzed by atomic attachment.

The findings reported here confirm that only the use of the unconventional method of GLCs, combined with high spatial–temporal resolution allows for achieving such detailed, atomic-resolution insights that make it possible to entangle the different growth mechanisms and to study their underlying mechanisms in detail. Therefore, our experimental approach confirms the previously suggested two-step nucleation mechanism and expand the current understanding of growth mechanisms based on atomic addition or/and particle attachment, by uncovering the detailed, atomic-scale mechanisms of classical and non-classical nucleation/crystallization theory. Thus, our study provides an in-depth insight into nanomaterial synthesis for the particular system investigated.

## Methods

A 5 mM solution of platinum precursor was prepared by dissolving Na_2_PtCl_4_·2H_2_O powder (> 99.9%) in deionized water (DI), which both were purchased from Sigma-Aldrich and used without further purification.

Two-type graphene coated TEM girds are purchased from Ted Pella, Inc: 300-mesh Cu TEM grids with a two-layer graphene film on a lacey carbon film and 300-mesh Cu TEM grids with a three-to-five layer graphene film on a lacey carbon film.

### Graphene liquid cell fabrication procedure

A step by step of fabrication process is shown in Fig. S1a. The GLC is prepared by a direct grid-on-grid sandwich technique^56^. Here are the details of how we carried out our graphene liquid cell:

The graphene coated TEM grids are washed with deionized water three times, and then placed with the graphene side up on a filter paper to dry. Afterward the grids are placed overnight on a pumping station before using them for cell preparation. Our cell fabrication starts by placing the two graphene-coated TEM grids with the graphene side up on a glass slide. Thereafter, we prepare a 5 mM solution of platinum precursor. Using a micropipette, we place a 0.5 µL droplet of the solution on top of the coated TEM grid, where a tweezers is used to hold the edge of the TEM grid down while placing the droplet so that the capillary forces do not pick up the TEM grid. When we are sure that the droplet is properly placed in the centre of the grid. The second TEM grid coated with two graphene layers is placed carefully with graphene side down on top of the droplet. Here, while a tweezers is used to hold its edge without touching the droplet, while placing the second grid. One edge of the top grid is placed down and then after the rest of the grid is placed gradually in order to avoid the stuck of the tweezers between the two grids. Then, finally, the second grid comes to rest on top of the first grid with no liquid being squeezed out. The graphene-coated TEM grids are pressed against each other for 30 min (time to ensure the formation of the liquid cell pockets). The capillary force between liquid and graphene brings the top and bottom graphene layers closer until the van der Waals (VdW) forces between the two layers dominate. Once the graphene layers on opposite sides adhere, pockets of trapped liquid form between the two grids, thus forming a GLC featuring a variety of liquid nanoreactors in terms of size, thickness, and amount of solution. As a last step of cell preparation, the grids are loaded on top of a single tilt TEM holder then transferred to a pre-vacuum system for 2 h, then this last is loaded into the column of an FEI Titan Themis 80–300 STEM. Figure S1b showing detailed successive steps in GLC fabrication process.

### Liquid-phase STEM experiments

The *in-situ* observations by liquid-phase STEM were carried out using an FEI Titan Themis 80–300 STEM with a probe Cs-corrector operated at 300 kV. ADF-STEM was routinely used, which provides a contrast approximately proportional to Z^*n*^ (with *n* = 1.6–1.8 and Z the atomic number), which is particularly useful for the study of heavy elements, such as Au, in a lighter matrix. The time series recorded in STEM were processed using ImageJ (Java 1.80_172 (64-bit) https://imagej.nih.gov/ij/) and DigitalMicrograph (Gatan) (Version 3.42.3048.0 https://www.gatan.com).

### Liquid pockets discussion

The presence of the aqueous solution inside the pockets during and after imaging is confirmed by the following observations.

1. Bubble formation: When the electron beam is focused on the liquid cells, bubbles can be seen around the Pt nanocrystals (Fig. S6). This is described in depth by previous studies on bubble formation, which is a very common phenomenon in liquid cell experiments^57–59^.
2. Irradiation effects: By irradiating different zones in the sample where there should be graphene layers, inside and outside of the pockets with the same electron dose (4.2 × 10^3^ electrons/Å^2^s). Contrary to the growth of Pt nanoparticles inside the liquid pocket, no obvious growth have been observed in the zone outside of the liquid pocket. This confirm the existence of the precursor solution in the formed pockets (more details in Fig. S7). Overall, these evidences indicate clearly that all the results in our experiments are obtained in liquid environments.
3. Nanoparticle motion: The nanoparticle motion in the membrane of the liquid pockets (Graphene layer) is different from that in solution. Pt nanoparticles on graphene layer always move via slow translation and rotation^16^, whereas nanoparticles in solution can translate and rotate freely. Figure S8 shows the existence of two types of particles; the first are stable (stacked to the graphene membrane) designed with red arrows and the second type is in motion inside the liquid pockets (white and green circles).

### In situ imaging conditions

Prior to insertion of the liquid cell, the TEM alignment were performed at 300 kV using an alignment sample.

During our study ADF-STEM imaging was performed by using a fixed probe size of 7 and condenser aperture (CA) 70 µm, in order to maintain constant the beam current when focalized on the liquid pockets and ensure an atomic resolution in liquid mode. We calculate the dose rate in electrons/Å^2^s by dividing the beam current by the surface irradiated by the beam, which is in the STEM mode advantageously corresponds to the imaged area. Therefore, the dose rate was easily controlled because it is inversely proportional to the square of the magnification. The electron dose rate used for imaging ranged from 80 to 4.2 × 10^3^ electrons/Å^2^s for magnification ranging from 90 k and 5 M. The time series were recorded with typical frame times of 0.5–2.0 frames/s and 200–500 frames/series. All the detailed acquisition parameters applied for recording the time series are summarized in Table S1.

## Supplementary Information

Supplementary Information.

Supplementary Video S1.

Supplementary Video S2.

Supplementary Video S3.

Supplementary Video S4.

Supplementary Video S5.

Supplementary Video S6.

Supplementary Video S7.
