# Supplementary material for: Multi-step atomic mechanism of platinum nanocrystals nucleation and growth revealed by in-situ liquid cell STEM
Source: Sci Rep. 2021 Dec 14;11:23965. doi: 10.1038/s41598-021-03455-w (PMC8671505; doi:10.1038/s41598-021-03455-w)
Supplement: Supplementary file 1 — Supplementary Information. [file 41598_2021_3455_MOESM1_ESM.pdf]

Supplementary Information

Multi-Step Atomic Mechanism of Platinum Nanocrystals Nucleation and Growth Revealed by In-Situ Liquid Cell STEM

Walid Dachraoui,*[¥] Trond R. Henninen,[¥] Debora Keller [¥] and Rolf Erni*[¥]

[¥]Electron Microscopy Center, Empa—Swiss Federal Laboratories for Materials Science and Technology, Überlandstrasse 129, CH-8600, Dübendorf, Switzerland.

*Correspondence to:
E-mail: walid.dachraoui@empa.ch
Rolf.erni@empa.ch

Table of contents

Supplementary Table: Tab. S1

Supplementary Figures: Figures S1 to S8

Captions for Movie S1 to S7

Tab. S1. Acquisition parameters used for the figures and the corresponding movies.

Movie	Probe / CA	Dose rate [e^{-}Å^{-2}s^{-1}]	Frame time [s]	Figure
Movie S1	7/70	300	0.25	Fig. 1
Movie S2	7/70	4.2 *10^3	0.5	Fig. 2 and Fig. S3
Movie S3	7/70	4.2 *10^3	0.5	Fig. S2
Movie S4	7/70	4.2 *10^3	0.5	Fig. 3
Movie S5	7/70	4.2 *10^3	0.5	Fig. 4a
Movie S6	7/70	4.2 *10^3	0.5	Fig. 4b
Movie S7	7/70	4.2 *10^3	0.5	Fig. 5b

Supplementary figures

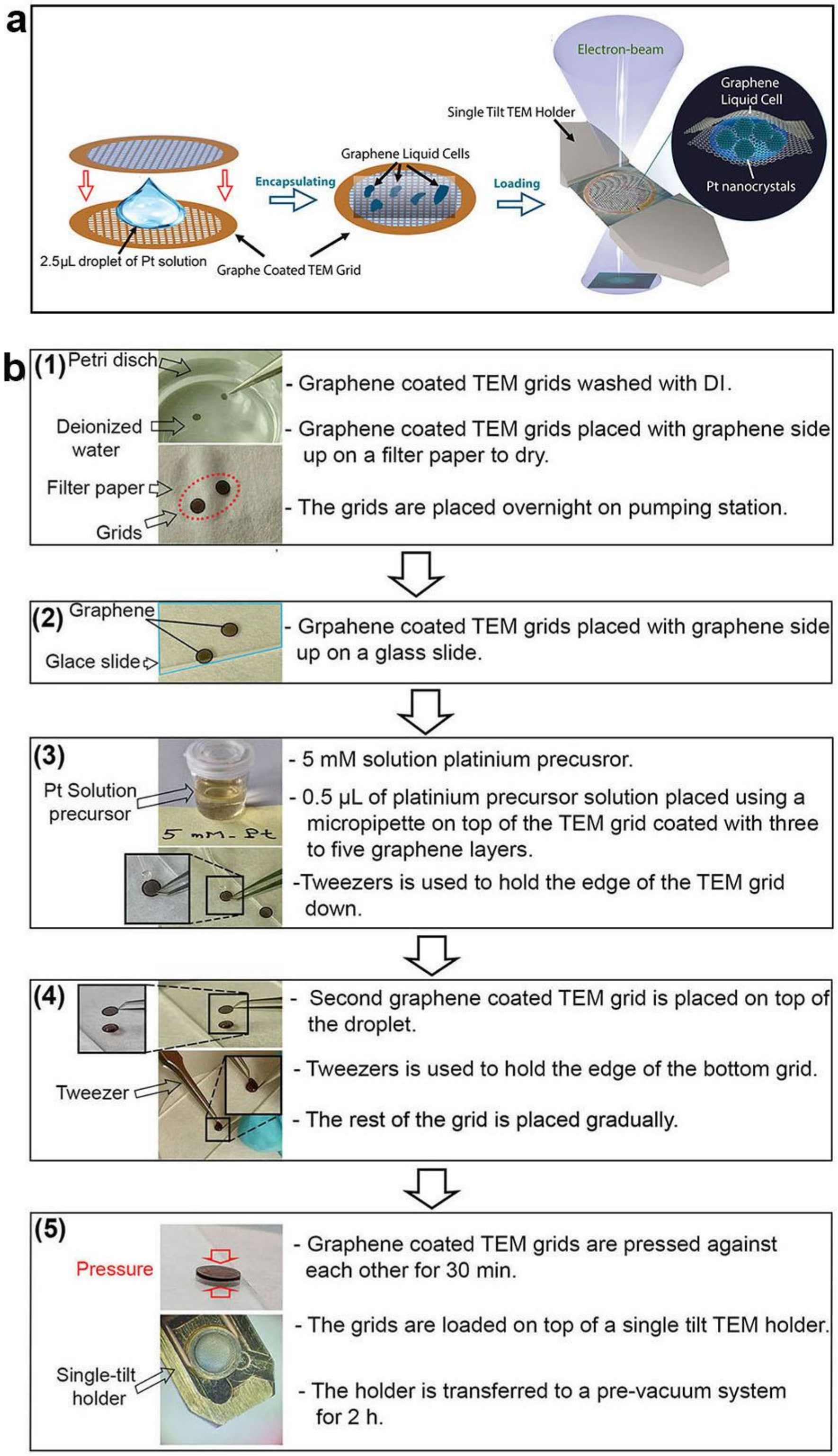

Figure S1. Step-by-step graphene liquid cell preparation using a direct grid-on-grid method. **a** A droplet of platinum solution is placed between two opposing graphene-coated grids. Removal of excess liquid eventually brings the two graphene layers in contact, thus forming sealed solution capsules between the two TEM grids. When s sealed solution capsules is form between the two graphene sheets, the TEM grids are transferred on top of a single tilt TEM holder, then inserted into the microscope. **b**. Schematic showing successive steps in GLC fabrication process.

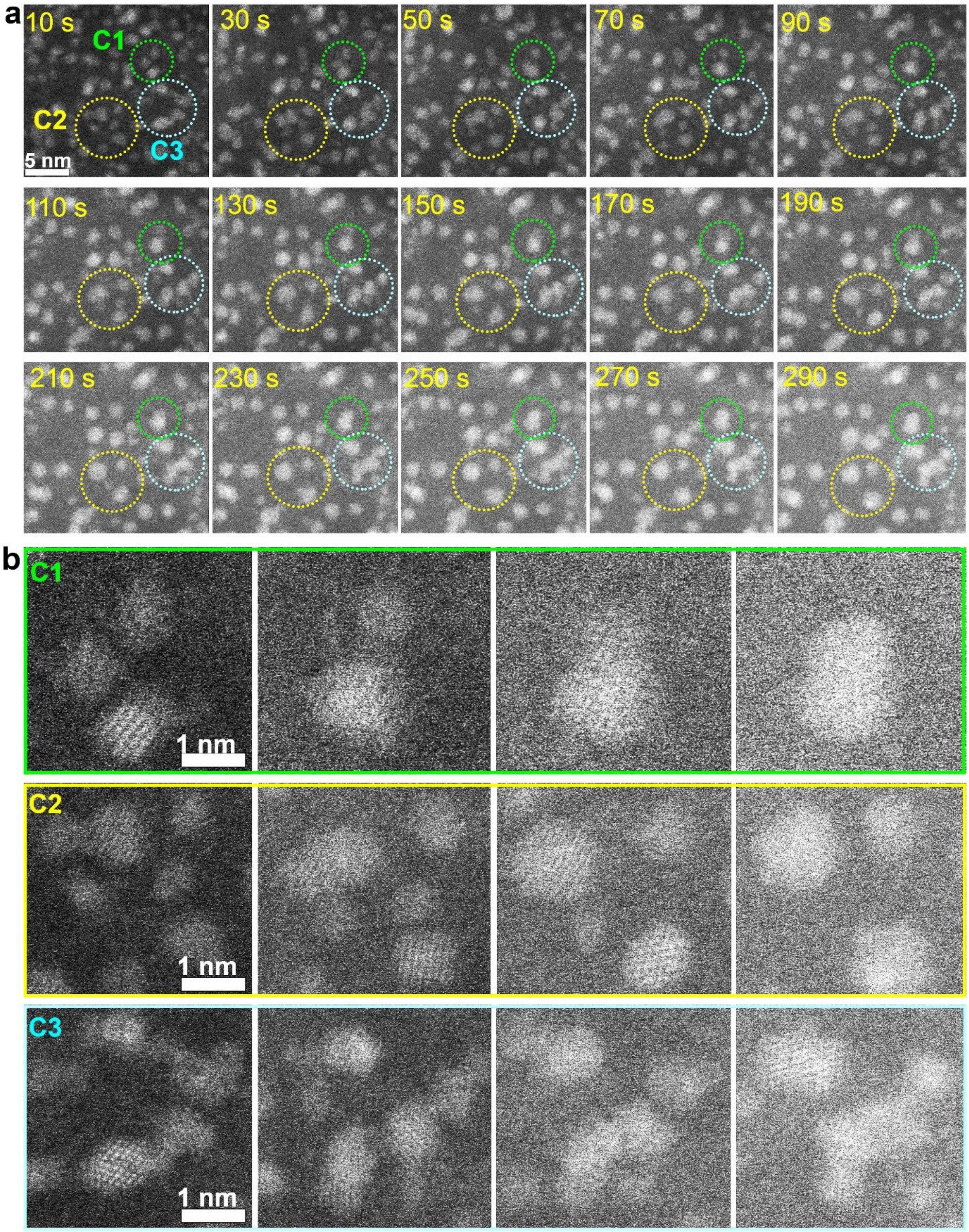

Figure S2. Pt nanocrystal coalescence in the second stage of growth. **a** ADF-STEM image sequence showing coalescence induced growth of Pt nanoparticles with three examples of coalescence C1, C2 and C3, highlighted with dashed circles (green, yellow and blue respectively). **b** Three ADF-STEM image sequences of the three coalescences C1, C2 and C3 revealing the coalescence processes at atomic-level.

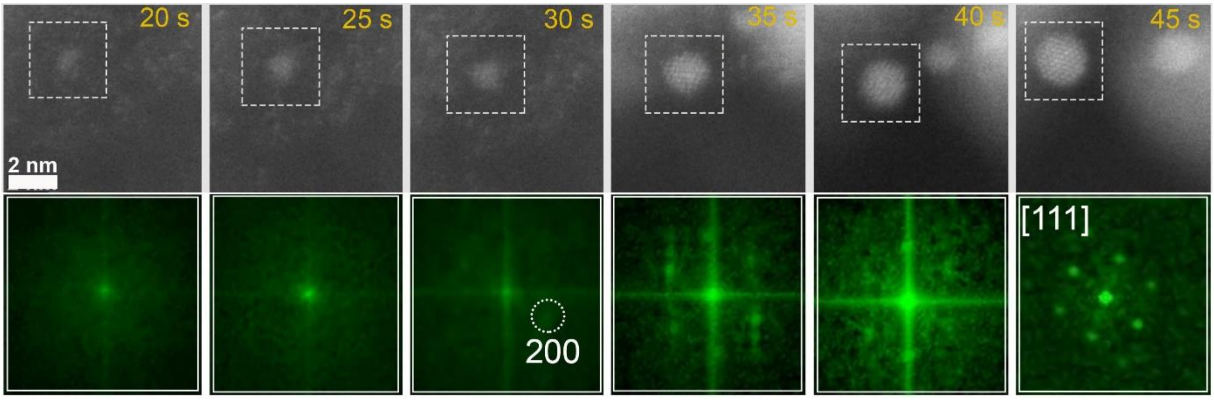

Figure S3. High-resolution ADF-STEM images showing the size evolution of a Pt nanocrystal in real-time and its corresponding FT patterns, which confirm the structural evolution, where the first nucleated cluster shows an FT pattern of an amorphous structure while at t = 60 s a first reflection appears corresponding to (200) planes of an fcc lattice. At the end, a well-crystallized nanoparticle is formed confirmed by the perfect [111] FT pattern of cubic platinum. Images of FFT patterns are shown in false colour.

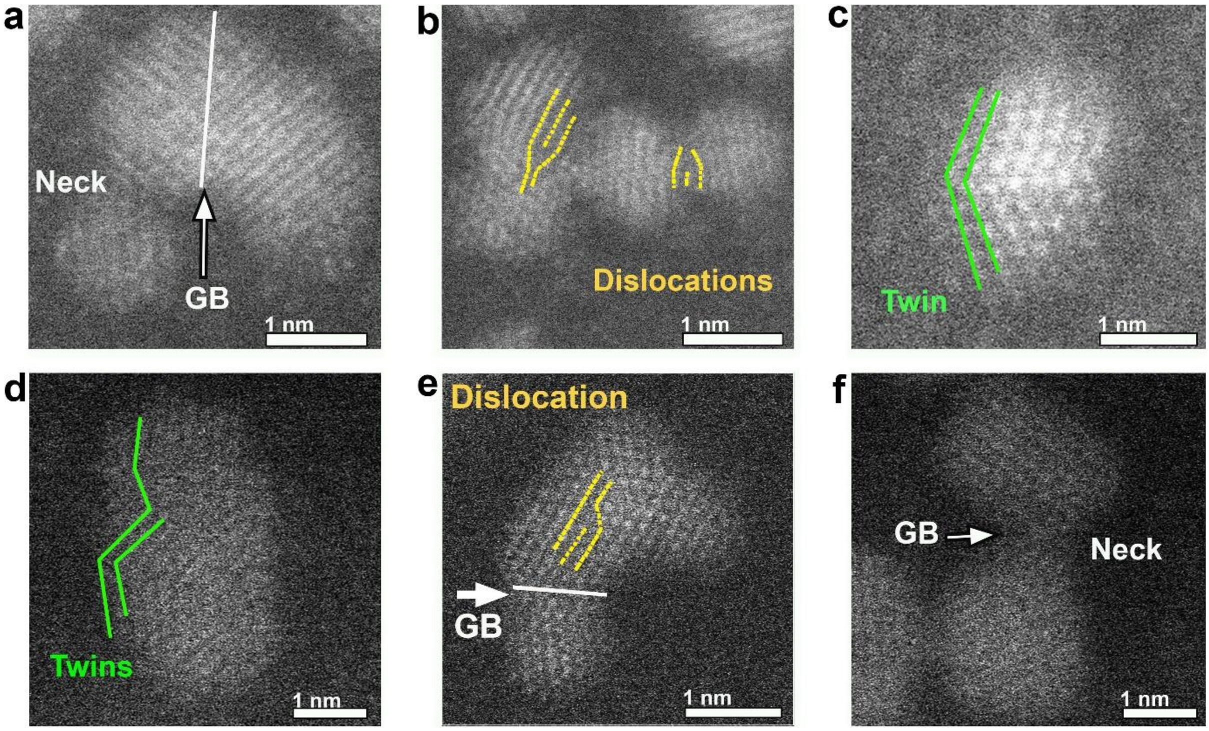

Figure S4. High-resolution ADF-STEM images of six examples of particles undergoing imperfect attachments, which induces defects at the interface. **a** Formation of a neck at the particle-particle interface and grain boundaries (GB). **b** Formation of dislocations. **c** and **d** Formation of twin boundaries. **e** Formation of dislocations and GB at the particle-particle interface. **f** Formation of a neck at the particle-particle interface and GB.

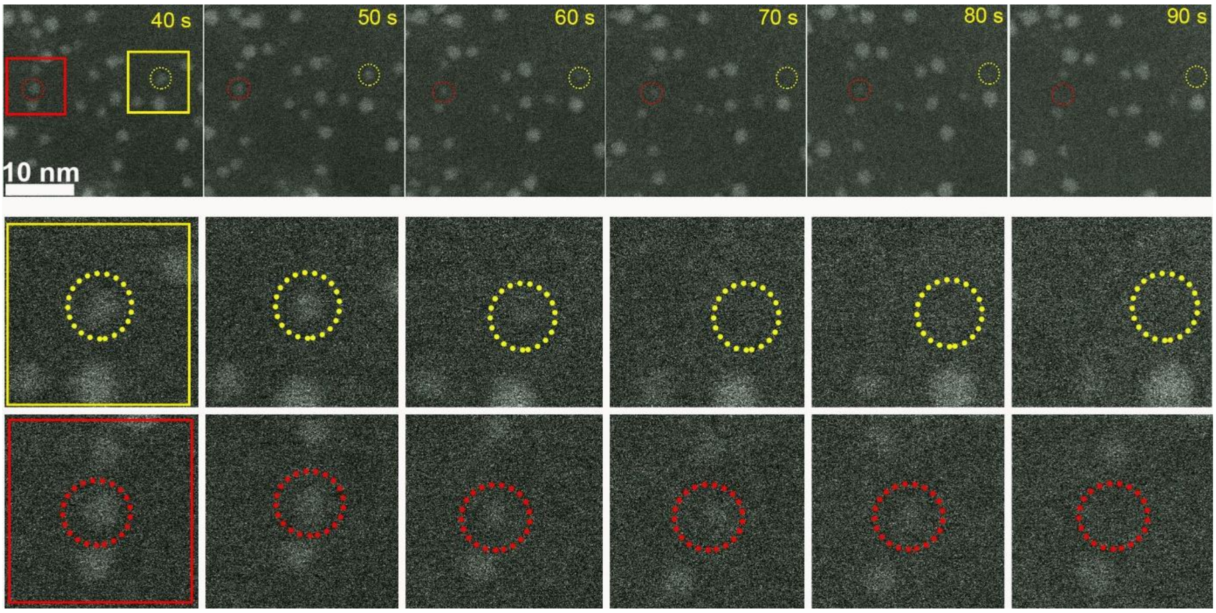

Figure S5. ADF-STEM images showing in real-time two examples of smaller particles dissolving in the surrounding of larger particles.Bottom series are a zoom-in corresponding to the particles highlighted with yellow and red squares in the top series. Images are shown in false colour.

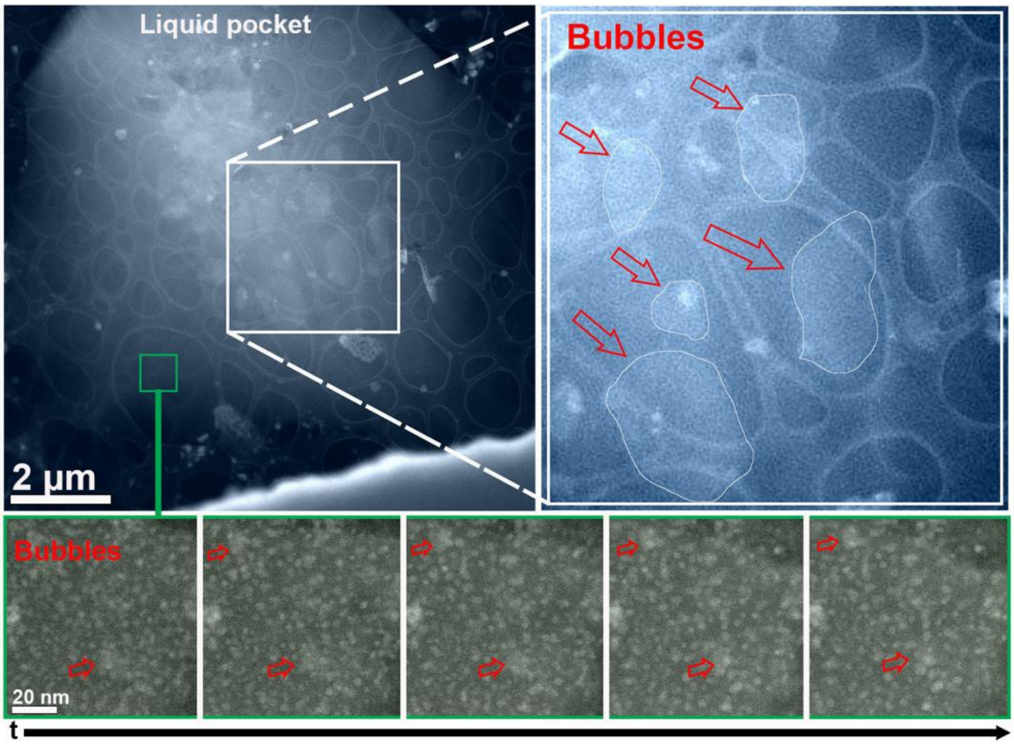

Figure S6: Representative low-magnification STEM image of the graphene liquid pockets showing the formation of bubbles. The right hand side image, shows a zoom-in of the zone designed by white square show the formed (highlighted with red arrows). In the bottom a series of HR-ADF-STEM images showing the formation of a bubble around nanocrystals.. Images are shown in false colour.

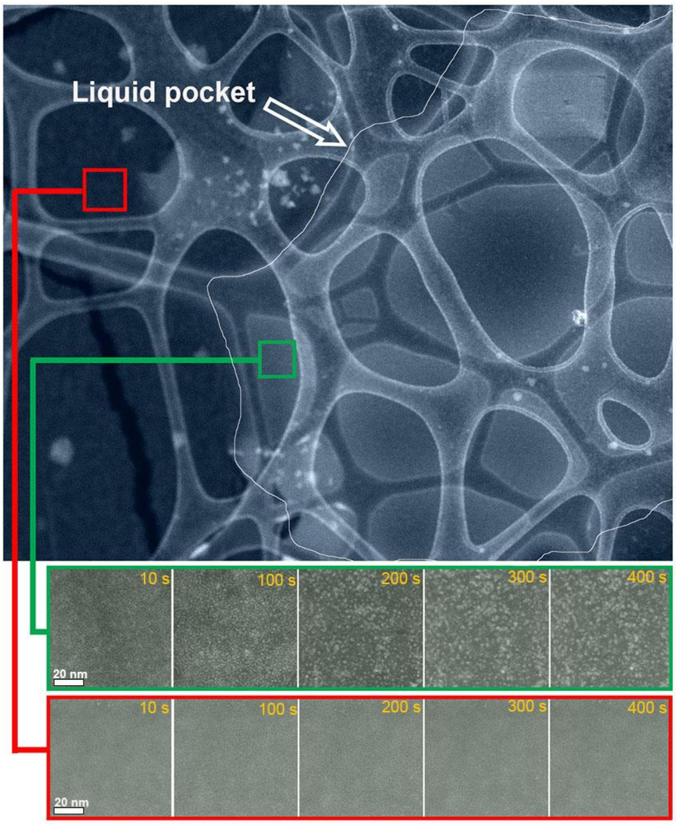

Figure S7: Top: Representative low-magnification TEM image of the graphene liquid pockets. **Red:** Series of HR-ADF-STEM images showing that there is no growth on the region outside of the liquid pocket. **Green:** growth of Pt nanocrystals inside the liquid pocket. Images are shown in false colour.

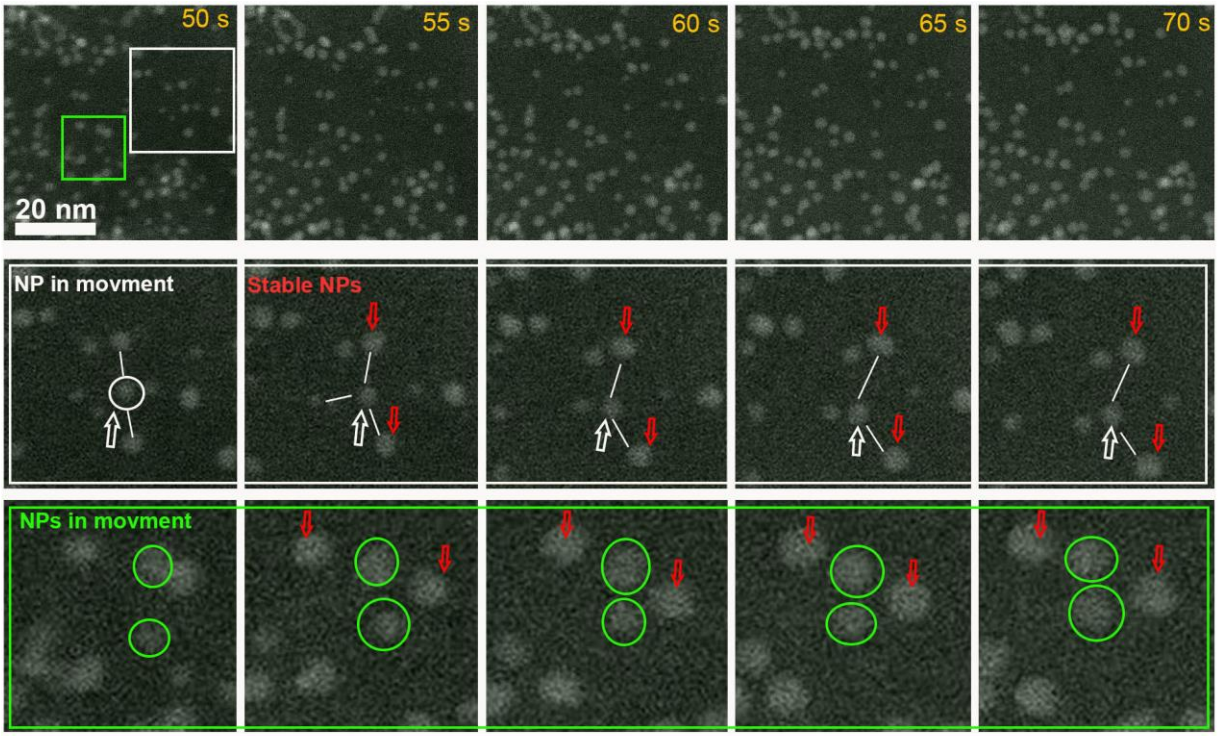

Figure S8: a. Representative time lapse HR-ADF-STEM imaging of movement of free nanocrystals in the liquid.. **b**. Zoom-in of the particles in the white square. c. Zoom-in of the particles in the white square. free nanocrystals dhighlighted with white and green circlesin c and d successively compared to the nanocrystals attached to the membrane (red arrows arrows). Images are shown in false colour.

Captions for movies

Movie S1: *In-situ* ADF-STEM observation of Pt nanoparticle growth in aqueous solution during exposure to the electron beam with an electron dose rate of about 4.2 10^3 electrons /Å^{2}s 300 electrons/Å^{2}s, as shown in figure 1 in the main text. The video plays at 10 x of the actual recording speed, scale bar 20 nm.

Movie S2: *In-situ* ADF-STEM observation of coalescence inducing a second stage of Pt nanocrystal growth in aqueous solution, with an electron dose rate of about 4.2 10^3 electrons/Å^{2}s, as shown in figure S2 in supporting information. The video plays at 5 x of the actual recording speed, scale bar 1 nm.

Movie S3: *In-situ* ADF-STEM observation of the reduction of Pt precursor with electron beam. Electron dose rate of about 4.2 10^3 electrons/Å^{2}s reducing Pt atoms, then condensation processes to form a Pt nanocrystal in aqueous solution and growth of Pt nanocrystal, as shown in figure 2 in the main text. The video plays at 1 x of the actual recording speed, scale bar 1 nm.

Movie S4: *In-situ* ADF-STEM observation at atomic level of a pair of Pt nanocrystals undergoing an oriented attachment process, with an electron dose rate of about 4.2 10^3 electrons/Å^{2}s, as shown in figure 3 in the main text. The video plays at 5x of the actual recording speed, scale bar 1 nm.

Movie S5: *In-situ* ADF-STEM observation at atomic level of a big Pt particle and three small clusters undergoing an imperfect attachment process completed with an oriented attachment process, with an electron dose rate of about 4.2 10^3 electrons/Å^{2}s, as shown in figure 4a in the main text. The video plays at 5 x of the actual recording speed, scale bar 1 nm.

Movie S6: *In-situ* ADF-STEM observation at atomic level of a typical twin boundary elimination process after an imperfect attachment growth, with an electron dose rate of about 4.2 10^3 electrons/Å^{2}s, as shown in figure 4b in the main text. The video plays at 5 x of the actual recording speed, scale bar 1 nm.

Movie S7: *In-situ* ADF-STEM observation at atomic level of typical big and small Pt crystals involved in an Ostwald ripening growth, with an electron dose rate of about 4.2 10^3 electrons/Å^{2}s, as shown in figure 5b in the main text. The video plays at 5 x normal speed, scale bar 1 nm.
